# A Fast EM Algorithm for BayesA-Like Prediction of Genomic Breeding Values

**DOI:** 10.1371/journal.pone.0049157

**Published:** 2012-11-09

**Authors:** Xiaochen Sun, Long Qu, Dorian J. Garrick, Jack C. M. Dekkers, Rohan L. Fernando

**Affiliations:** 1 Department of Animal Science and Center for Integrated Animal Genomics, Iowa State University, Ames, Iowa, United States of America; 2 Department of Statistics, Iowa State University, Ames, Iowa, United States of America; Pennsylvania State University, United States of America

## Abstract

Prediction accuracies of estimated breeding values for economically important traits are expected to benefit from genomic information. Single nucleotide polymorphism (SNP) panels used in genomic prediction are increasing in density, but the Markov Chain Monte Carlo (MCMC) estimation of SNP effects can be quite time consuming or slow to converge when a large number of SNPs are fitted simultaneously in a linear mixed model. Here we present an EM algorithm (termed “fastBayesA”) without MCMC. This fastBayesA approach treats the variances of SNP effects as missing data and uses a joint posterior mode of effects compared to the commonly used BayesA which bases predictions on posterior means of effects. In each EM iteration, SNP effects are predicted as a linear combination of best linear unbiased predictions of breeding values from a mixed linear animal model that incorporates a weighted marker-based realized relationship matrix. Method fastBayesA converges after a few iterations to a joint posterior mode of SNP effects under the BayesA model. When applied to simulated quantitative traits with a range of genetic architectures, fastBayesA is shown to predict GEBV as accurately as BayesA but with less computing effort per SNP than BayesA. Method fastBayesA can be used as a computationally efficient substitute for BayesA, especially when an increasing number of markers bring unreasonable computational burden or slow convergence to MCMC approaches.

## Introduction

Genomic prediction of breeding values for economically important traits of farm animals based on high-density genome-wide SNP genotypes is typically performed in two steps [1]. First, allele substitution effects of SNPs are estimated from a reference population with both trait phenotypes and SNP genotypes (training); then, the genomic estimated breeding values (GEBV) for selection candidates, often the genotyped progeny of the training population, are obtained by summing the estimated SNP effects across the genome [1], [2]. In this second step, which in a research context we refer to as validation, the prediction accuracy of GEBV can be assessed by the correlation of GEBV with either true breeding values (TBV) or phenotypes. Comparative studies on both simulated and field data have shown that GEBV tend to have higher accuracy than breeding values estimated using pedigree relationships [2], [3], depending on the genetic architecture of the trait [4], the nature of the SNP panel [1], [5], [6], the size of the training data [6]–[8], the population structure [9] and the relationship between training and validation individuals [3], [10].

Currently, two classes of methods are used to overcome the over-parameterization problem of linear models used for genomic prediction when relating a lesser number of phenotypes to a larger number of SNP genotypes. The first is best linear unbiased prediction of SNP effects from a linear mixed model in which random SNP effects are assumed to be independently and identically distributed as zero-mean normal random variables with a common effect variance (ridge regression) [1], [3]. This corresponds to an assumed genetic architecture characterized by a large number of loci contributing equally to the overall genetic variance of the trait. The model for ridge regression is equivalent to an animal model in which a marker-derived realized relationship matrix is used as the variance-covariance structure of random genomic breeding values (GBLUP) [3], [7], [11]. Equation 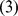 of Habier et al. [3] showed that the expected covariance between marker genotypes of two individuals is proportional to the additive relationship coefficient among them. Assuming variance components known, solving for SNP effects as linear combination of best linear unbiased predicted breeding values from GBLUP can be efficient because the dimension of mixed model equations for GBLUP is the number of individuals, which is usually much smaller than the number of SNPs [12]. The second class of methods for genomic prediction do not necessarily result in prediction rules that are linear in the observed phenotypes. These methods are often based on Bayesian hierarchical models and are implemented through Markov chain Monte Carlo (MCMC) sampling, for instance, BayesA [1], BayesB [1], Bayesian LASSO [13], [14], BayesC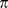 [15], etc. Prior distributions for SNP effects are chosen to shrink ignorable small effects towards zero. Sampled SNP effects are averaged over MCMC iterations to obtain posterior means of SNP effects. Depending on the choice of priors, most Bayesian hierarchical methods impose stronger shrinkage towards zero on small SNP effects and less shrinkage on relatively large effects by allowing each SNP to have a distinct effect variance (e.g. BayesA) and/or by fitting a mixture distribution that assumes any SNP might come from a continuous distribution or a distribution degenerate at zero (e.g. BayesB). The mixture fraction is influenced through a hyperparameter 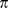, which specifies the prior proportion of SNPs that have zero effects. At the cost of higher computing effort, Bayesian methods tend to achieve higher prediction accuracy than GBLUP for simulated datasets [1]–[3], [16]. Further, results from real data often show that methods that fit all SNPs in the model (GBLUP and BayesA) tend to give similar accuracy as methods with variable selection, suggesting that most economically important traits might be controlled by a large number of loci with relatively small effects [8], [10], [17], [18].

Several non-MCMC algorithms have been proposed to improve computational efficiency for linear models with differential shrinkage of SNP effects and/or with variable selection. VanRaden [7] presented two non-linear predictions A and B that are analogous to BayesA and BayesB in Meuwissen et al. [1], respectively. The ratio of residual variance over common effect variance in ridge regression, which controls the amount of shrinkage of SNP effects, is modified depending on the size of estimated SNP effects to allow differential shrinkage. Estimates of SNP effects are calculated efficiently using Jacobi iteration. Both simulation [7] and real data [19] showed that VanRaden [7] non-linear predictions were fast and accurate for large datasets. Moreover, Expectation-Maximization (EM) algorithms [20] can in some cases be computationally more efficient than MCMC approaches. Bayesian LASSO, which uses a double exponential (DE) prior distribution for SNP effects, and BayesA, which assumes 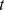 prior distribution for SNP effects, have been adapted to fast non-MCMC deterministic or EM algorithms. Meuwissen et al. [21] presented a fast heuristic iterative conditional expectation (ICE) algorithm, where the posterior expectation of SNP effects was calculated analytically, assuming a fixed known DE parameter and dispersion parameters. Shepherd et al. [22] formulated an EM algorithm which they called emBayesB, based on the same model as ICE, which used an indicator variable for each SNP that is in linkage disequilibrium (LD) with QTL as missing data, and estimated SNP effects and the DE parameter in the M-step. Yi and Banerjee [23] derived an EM algorithm for a BayesA model for QTL detection by treating the unknown SNP effect variances as missing data. Hayashi and Iwata [24] developed a generalized EM algorithm (EM-BSR) with a slightly different M-step and further extended it to a heuristic algorithm for the BayesB model. BayesA modeling of SNP effects can be more appealing than LASSO, in that the estimated SNP effects are nearly unbiased for large effects, while in LASSO the bias does not diminish even when SNP effects are large [25].

In this study we formulate a principled EM algorithm (termed “fastBayesA”) that converges to a joint posterior mode of SNP effects under the BayesA model. By applying the method to simulated datasets with contrasting sizes and genetic architectures, fastBayesA is shown to predict GEBV as accurately as BayesA but with less computing effort per SNP than BayesA. The latter will become more important as SNP densities increase to that provided by individual DNA sequence.

## Materials and Methods

### Statistical Model

The linear mixed model for phenotypes based on GBLUP is
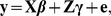
where 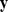 is an 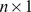 vector of phenotypes, with 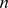 equal to the number of individuals in the training dataset; 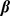 is a vector of fixed effect parameters and 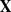 is a known design matrix relating fixed effects to phenotypes; 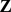 is an 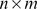 matrix of SNP genotypes in the “
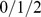
” allele dosage coding, with row 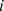 containing genotypes of 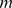 SNPs for individual 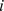; 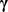 is an 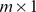 zero-mean random vector of allele substitution effects with 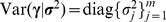, where 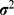 is an 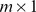 vector with the 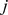
th element 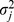 being the effect variance of SNP 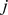; and 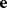 is an 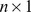 vector of independently and normally distributed random errors with mean 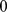 and variance 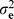. In Meuwissen et al. [1], GBLUP assumes that effect variances 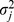 are known and the same for all SNPs and that the SNP effects are *marginally* normally distributed, whereas BayesA assumes a scaled inverse Chi-square prior distribution for effect variances with scale parameter 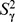 and degrees of freedom 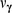, and a normal distribution for the effect of SNP 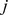 *conditional* on its variance, i.e.,



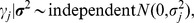
where 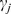 is the 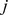
th element of 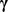, and



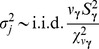
for all 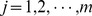. It can be shown that in BayesA the marginal distribution of the SNP effect is scaled univariate-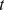 with degrees of freedom 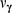 and scale parameter 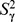 [26].

### Efficient Solving of SNP Effects Using an Equivalent Animal Model

The calculation strategy to develop fastBayesA follows Strandén and Garrick [12] and is generalized here. The phenotype can be modeled by the following animal model [27]:
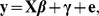
where 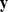, 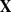, 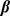 and 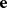 are as previously defined, 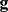 is an 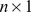 vector of genomic breeding values of the individuals, which can be modeled as the sum of the 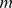 SNP effects, as described above, i.e., 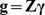. This genomic animal model is equivalent to the GBLUP model given normality of SNP effects. The (co)variance matrix of genomic breeding values is



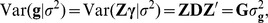
where 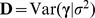, 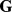 is the realized relationship matrix derived from the SNP genotypes and 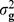 is the variance of genomic breeding values. Element 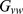 of 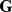 is the proportion of SNPs that are IBD between individuals 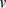 and 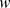 [28], [29]. For GBLUP, the common effect variance of SNPs is equal to 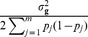 in which 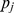 is the minor allele frequency of SNP 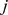 [3]. Given 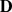, the BLUP 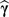 of SNP effects 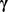 can be efficiently computed in two steps using the animal model [12]. First the BLUP of genomic breeding values 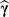 is obtained by solving the mixed model equations of the animal model, then 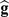 can be solved following Strandén and Garrick [12] as:



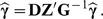



### EM Algorithm for Estimating SNP Effects

We use the above relationships to develop an EM algorithm for BayesA by treating the effect variance of each SNP as missing data. In the E-step, the conditional expectation of the logarithm of the joint probability of 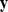, 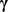 and 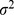, with expectation taken over the distribution of 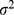 conditional on the observed phenotypic data 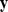 and the current estimate (the 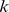
th step) 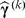 of SNP effects, is calculated:
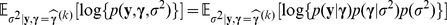


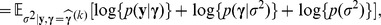
where we use the shorthand notation 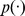 to represent the marginal density of  and 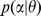 notation represents the conditional density of 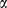 given 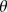. The first term of this expectation is free of 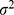. The second term of the expectation is over the sum of the logarithms of normal densities for 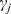 and can be calculated element-wise. And the third term is free of 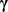. Hence



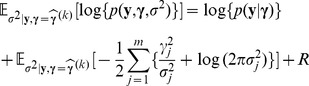


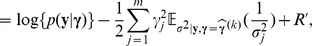
where 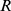 and 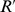 are the remaining terms that are free of 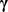. As shown in Appendix S1, the conditional distribution of 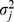 given 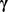 is a scaled inverse Chi-square distribution with degrees of freedom 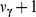 and scale parameter 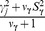, and



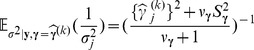
and



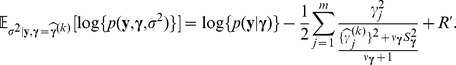



The M-step of the algorithm is to maximize the above expectation with respect to 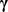, which is equivalent to finding the BLUP of SNP effects as described in the previous section, using 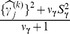 as effect variance for SNP 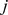, i.e., the 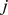
th diagonal element of 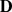. After iterating between the E-step and the M-step until convergence, a local posterior mode of 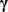 will be obtained. Details of the maximization and the estimation equations are shown in Appendix S2. Because of the success of GBLUP in traditional breeding methods, we choose the starting values for 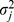 to be the variance under the GBLUP method, i.e. 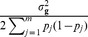, where 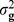 is the genetic variance, which will be assumed known in simulation.

### Simulation

Prediction of breeding values and computational efficiency of fastBayesA were compared to other methods by applying to simulated phenotypes and SNP genotypes of pedigreed populations. The initial generation comprised a population of effective size 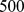 that was randomly mated for 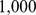 generations to reach mutation-drift equilibrium and then gradually expanded to an actual size of 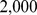 in the next 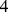 generations. In the 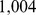
th generation, 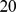 sires and 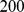 dams were randomly sampled without replacement from the 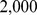 individuals in generation 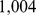 to represent the founders of the pedigree. Each of the 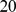 sires in these and subsequent generations was randomly mated to 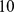 different dams, with each dam producing 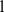 male and 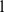 female offspring. That scheme continued for several generations at a constant size of 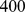 (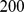 male and 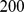 female offspring).

Two datasets were generated for the comparison of alternative methods in terms of prediction accuracy of GEBV (Dataset A) and computing time (Dataset B). Dataset A includes four scenarios of different genetic architectures and Dataset B varies in training size and genome length. The scenarios used in each dataset are summarized in Table 1. The standard scenario was a training group of 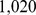 individuals from the first three pedigree generations, two chromosomes with 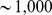 SNPs each, and a total number of 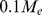 QTL, (A1 and B2 of Table 1), where 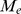 is the number of independently segregating loci across the genome, computed following Goddard [30] and Hayes et al. [28] and is given in Table 1 for the different scenarios. SNP loci and QTL were sampled among simulated loci to have minor allele frequency larger than 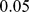. For scenario B1, B2 and B4, the first 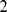, 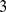 and 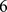 pedigree generations were used for training, respectively, and the five generations following training were used for validation.

**Table 1:** Summary of simulated datasets and scenarios.

Dataset	Dataset A				Dataset B				
Scenario	A1	A2	A3	A4	B1	B2	B3	B4	B5
Training size	1,020				620	1,020	1,020	1,020	2,220
No. chromosomes	2				2	2	5	10	2
*M_e_*	241				241	241	543	1,010	241
No. QTL	0.1*M_e_*	0.1*M_e_*	2.0*M_e_*	2.0*M_e_*	0.1*M_e_*				
QTL variance	hetero	const	hetero	const	hetero				

Scenarios differed in training data size, number of chromosomes, number of QTL, and whether the genetic variance contributed by QTL was constant (const) or heterogeneous (hetero).

Each chromosome was 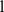 Morgan in length and initially evenly covered by 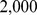 SNPs, among which 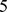 times the desired number of QTL were randomly positioned as candidate QTL to guarantee enough QTL segregating at mutation-drift equilibrium. The SNPs and QTL were biallelic, with initial allele frequencies 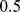 and in Hardy-Weinberg equilibrium. Mutation rate was 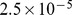 per meiosis per locus for both QTL and SNPs. The number of crossovers per chromosome was sampled from a Poisson distribution with mean 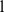. Recombination rates were modeled by the Haldane mapping function [31]. At generation 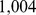, all SNPs with minor allele frequency less than 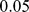 were eliminated and the desired number of QTL were randomly selected from candidate QTL with minor allele frequency larger than 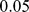. QTL effects were generated according to different scenarios and scaled to achieve a total genetic variance of 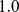 in generation 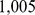. In scenarios where QTL variances were heterogeneous, QTL effects were randomly sampled from a Gamma distribution with shape parameter 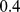 and scale parameter 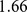 [1], while in scenarios with constant QTL variances, the effect of the 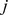
th QTL was backsolved as the square root of 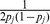, with equal probability of being positive or negative, where 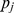 is the minor allele frequency at generation 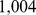.

True breeding values were obtained by summing up all QTL effects for a given individual. In Dataset A, normal random errors with mean 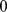 and variance 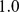 or 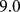 were added to true breeding values to generate phenotypes of traits with heritability 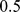 or 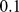, respectively. The simulated heritability for all scenarios in Dataset B was 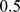. For each scenario, these activities were repeated to provide 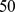 replicates. All replicates used the same initial SNP positioning but varied in the position of QTL and SNPs and in the effects of QTL after selecting loci with minor allele frequencies larger than 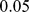.

For the analysis of the simulated datasets using the Bayesian methods, the degrees of freedom of the prior distribution for effect variance and residual variance was 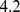, following Meuwissen et al. [1]. BayesA and BayesB were implemented in genomic selection software GenSel [32]. Formulation of BayesA and BayesB was almost identical with Meuwissen et al. [1] except that the effect of each SNP instead of haplotype was sampled by MCMC in GenSel. The proportion of the number of QTL over the total number of SNPs was used for 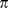 in BayesB. Simulated variance components were provided to the mixed model equations in fastBayesA and used to estimate hyperparameters of prior distributions for variance components.

For Bayesian methods, the MCMC was run for 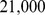 iterations, with the first 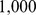 discarded as burn in. The fastBayesA algorithm stopped when the change of estimated SNP effects became small, i.e.
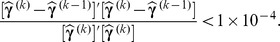



## Results

### Prediction Accuracy and Bias of GEBV under Alternative Genetic Architectures

Eight scenarios of contrasting heritability, number of QTL and distribution of QTL variance were simulated to represent a range of genetic architectures. The average correlation and regression coefficient of TBV on GEBV in the first validation generation from 50 replicates are shown in Table 2. Method fastBayesA had similar accuracy to BayesA and was much more accurate than GBLUP but less accurate than BayesB, regardless of genetic architecture or heritability. The results are as expected, in that fastBayesA predicts GEBV with similar accuracy as BayesA.

**Table 2:** Accuracy of GEBV and regression coefficient of TBV on GEBV in the first validation generation of Dataset A for GBLUP, BayesA, BayesB and fastBayesA.

Heritability	0.5				0.1			
No. QTL	0.1*M_e_*		2.0*M_e_*		0.1*M_e_*		2.0*M_e_*	
QTL Variance	Hetero^1^	Const^2^	Hetero	Const	Hetero	Const	Hetero	Const
Accuracy of GEBV								
GBLUP	0.777^3^	0.777	0.765	0.749	0.516	0.511	0.509	0.470
BayesA	0.832	0.834	0.778	0.764	0.552	0.543	0.515	0.477
BayesB	0.869	0.866	0.789	0.777	0.598	0.593	0.522	0.486
fastBayesA	0.839	0.841	0.777	0.763	0.544	0.539	0.509	0.476
Regression coefficient of TBV on GEBV								
GBLUP	0.979^4^	0.981	0.984	0.968	0.953	0.949	0.954	0.888
BayesA	0.947	0.955	0.985	0.976	0.942	0.952	0.956	0.901
BayesB	1.019	1.009	0.996	0.991	1.050	1.083	0.964	0.932
fastBayesA	0.902	0.905	0.887	0.873	0.887	0.891	0.906	0.867

1. Heterogeneous genetic variance of QTL.

2. Constant genetic variance of QTL.

3. Mean of correlation of TBV with GEBV over 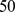 replicates. Standard errors were less than 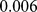 for all scenarios with heritability 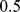 and less than 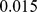 for scenarios with heritability 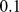.

4. Mean of regression coefficient of TBV on GEBV over 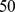 replicates. Standard errors were less than 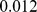 for all scenarios with heritability 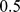 and less than 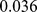 for scenarios with heritability 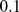.

As the number of QTL increased from 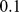 to 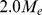, the accuracy of (fast)BayesA and BayesB decreased by up to 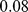, while that of GBLUP did not drop as much. This result is in accordance with Daetwyler et al. [4], in that the accuracy of GBLUP was not affected by the number of QTL. However, even when the number of QTL was 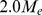, the accuracy of the Bayesian methods remained higher than that of GBLUP, which contradicts Daetwyler et al. [4], who found that the advantage of BayesB over GBLUP diminished as the number of QTL increased up to 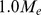. The contradiction was probably due to the fact that the training size relative to genome length was much larger in our study than in Daetwyler et al. [4].

Bias in the prediction of GEBV is shown by the deviation of regression coefficients from 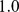 in Table 2. Except for BayesB, which had regression coefficients close to 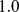, regression coefficients were substantially below 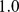 for the other methods, as low as 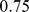. In all scenarios, the regression coefficients for fastBayesA were smaller than those for BayesA, indicating larger bias of fastBayesA than BayesA in predicting TBV. This suggests that the estimated SNP effects and hence GEBV are not shrunk enough. The reason might be that the joint posterior mode of SNP effects, which is obtained as the estimate in fastBayesA, can deviate substantially from the posterior means used in BayesA due to the asymmetry of the posterior densities. An improper scale of the genomic relationship matrix could also result in biased GEBV.

### Decline of Accuracy Over Generations

**Figure 1:**
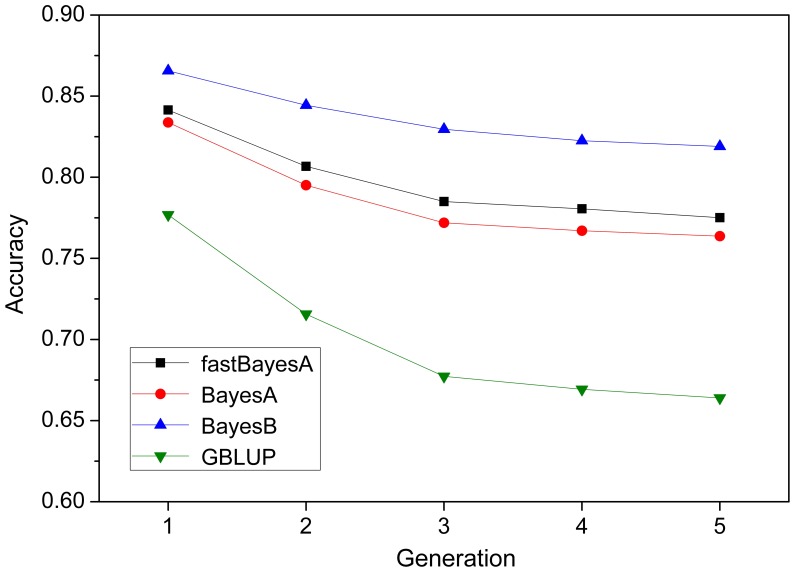
Prediction accuracy of GEBV in five validation generations by alternative methods. The scenario is 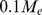 QTL with heterogeneous variance, heritability 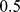. Results are averaged over 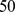 replicates.

Figure 1 shows the mean prediction accuracy of GEBV in five consecutive generations after training in the scenario with heritability 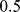 and 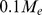 QTL with equal variance. For all four methods, accuracy decreased with generations, in agreement with Habier et al. [3]. The accuracies of fastBayesA and BayesA were very similar in all five generations and were higher than accuracies of GBLUP and lower than accuracies of BayesB. The decrease in accuracy over the five generations was largest for GBLUP and smallest for BayesB, with (fast)BayesA in between. Similar trends were also observed in other scenarios with different genetic architectures (results not shown).

#### Accuracies across EM iterations

**Figure 2:**
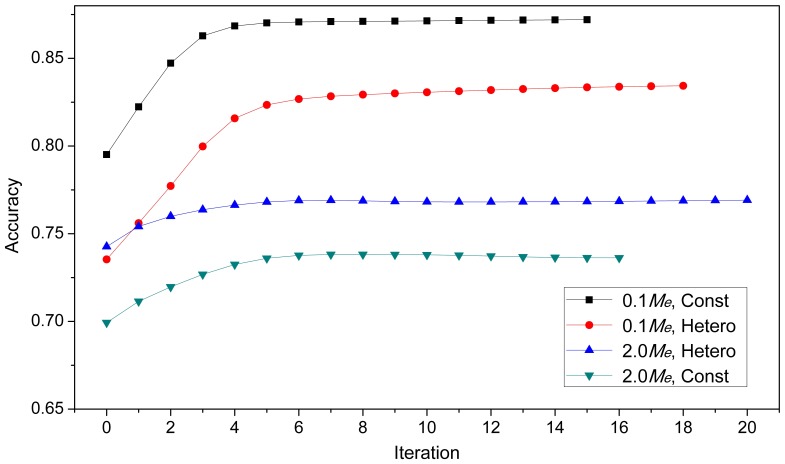
Prediction accuracies of GEBV across EM iterations in the first validation generation. The four scenarios are 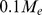 QTL with constant variance (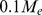, Const), 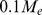 QTL with heterogeneous variance (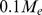, Hetero), 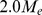 QTL with heterogeneous variance (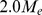, Hetero) and 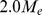 QTL with constant variance (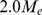, Const). Results for each scenario are averaged over 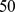 replicates.

To study the optimizing property of fastBayesA, accuracies of GEBV in the five validation generations were calculated at each EM iteration until the convergence criterion was achieved. Figure 2 shows the accuracy at each iteration in the first validation generation from one random replicate of each scenario in Dataset A (heritability was 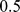). The accuracy of GEBV from fastBayesA increased gradually with iteration and stabilized at a higher accuracy than GBLUP, which is the accuracy achieved in the first iteration. In Figure 2, the accuracy stabilized within 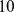 steps but the algorithm continued for several more steps before reaching the convergence criterion, which was based on changes in estimated SNP effects rather than estimated breeding values. This indicates that the accuracy of GEBV is insensitive to small changes in SNP effects.

#### Computational efficiency of EM

Computational efficiency of different methods was compared in relation to training population size and size of SNP panels. Results are in Table 3. Method fastBayesA has less computing effort per SNP than BayesA. The increase in computation time is likely to be between quadratic to cubic with the number of individuals, depending upon the actural algorithm used for solving the mixed model equations.

**Table 3:** Computing time (in seconds) for training by BayesA, BayesB and fastBayesA.

Training size	620	1,020	1,020	1,020	2,220
No. chromosomes	2	2	5	10	2
BayesA	321.7	479.8	1,215.2	2,492.8	928.8
BayesB	376.8	473.7	1,194.0	2,384.5	687.9
fastBayesA	25.3	63.0	114.6	168.2	350.5

## Discussion

In this study, a fast EM algorithm fastBayesA was developed for genomic selection without MCMC. The method is non-stochastic, but only approximates BayesA estimates of marker effects and GEBV because it uses a joint posterior mode of effects rather than the posterior means used in BayesA. Compared with MCMC-based Bayesian methods on the simulated datasets, fastBayesA was shown to have similar prediction accuracy to BayesA but less computational effort per SNP than BayesA.

An EM algorithm with the marginal distribution of SNP effects modeled as a 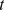 distribution was first proposed by Yi and Banerjee [23] for mapping QTL with epistatic and genotype-by-environment interaction effects. Since their main objective was to map major QTL, they used few degrees of freedom and a small scale parameter for the inverse Chi-square prior for the effect variance, which imposed heavy shrinkage on small effects such that only large effects would be detected. This is not ideal for genomic prediction for which many SNPs with small effects can usefully contribute to predictions in models influenced by polygenic gene action. Based on the same EM formulation as Yi and Banerjee [23], Hayashi and Iwata [24] presented a generalized EM algorithm (EM-BSR) for genomic prediction, but in the M-step only partial maximization is performed. The method fastBayesA that was developed in this study, following Yi and Banerjee [23], was also designed for predicting breeding values but has a different formulation than EM-BSR in the maximization step. In fastBayesA, the posterior distribution of SNP effects was jointly maximized using BLUP, which is more efficient and requires fewer EM iterations to converge. The advantage of the M-step of fastBayesA is that all SNP effects can be estimated simultaneously and computational efficiency is insensitive to the number of SNPs.

The computational efficiency of fastBayesA is sensitive to the number of individuals in training since construction and inversion of the realized relationship matrix is computationally expensive. For datasets with a large number of training individuals, the faster Jacobi iteration as in VanRaden [7] can be used to obtain the BLUPs of SNP effects in fastBayesA. Since computing time of the Bayesian MCMC methods is expected to increase linearly with the number of markers, fastBayesA can be advantageous over MCMC-based methods as marker density increases, as it will until all polymorphisms available from whole genome resequencing are used as candidates.

Both in BayesA and fastBayesA, inferences are based on the same posterior distribution that may not be unimodal, and both methods have to be used with caution. In BayesA the posterior mean is used to estimate SNP effects, and when the marginal posterior distribution for SNP effect is multimodal, the MCMC sampler will tend to stay within the neighborhood of a local mode and fail to visit other modes that are distant from this one [33]. Therefore, the empirical distribution from the MCMC samples may be different from the true posterior distribution and the posterior mean estimated by MCMC samples may not be accurate. In fastBayesA a joint posterior mode is used to estimate SNP effects, and the mode that the EM algorithm finds may not be the global mode. The GBLUP estimates of SNP effects provide a reasonable starting point that guarantees fastBayesA estimates will at least be no worse than GBLUP estimates.

**Figure 3:**
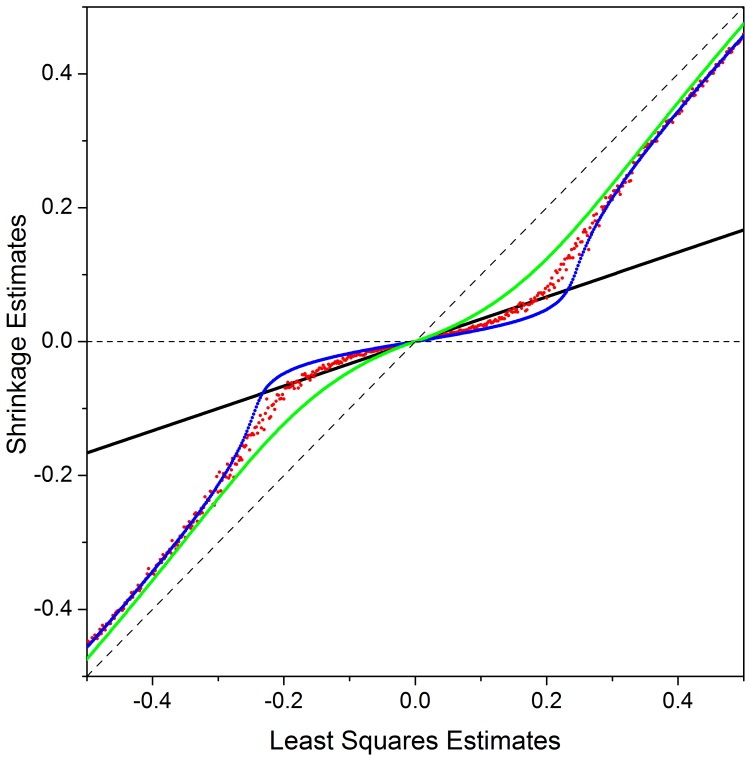
Shrinkage estimate of SNP effects from ridge regression (black line), BayesA (red dots), fastBayesA (blue line) and VanRaden non-linear prediction A (green line) against least squares estimate. SNP effects are measured in standard deviation units.

**Figure 4:**
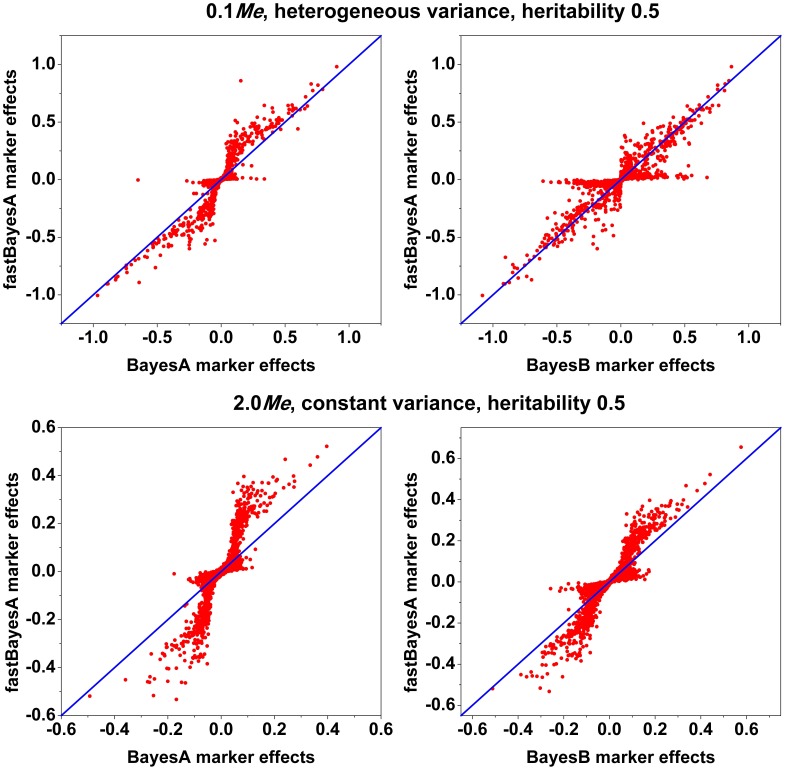
Estimated SNP effects from fastBayesA (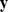 axis) against estimates from BayesA and BayesB (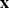 axis). All SNPs across 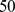 replicates are pooled for each scenario. Red dots show estimated SNP effects, and the blue line represents 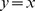.

Method fastBayesA results in similar prediction accuracy as BayesA because of their identical modeling of SNP effects. Any differences in accuracy are due to the fact that the joint posterior mode of SNP effects used in fastBayesA can be quite different from the posterior means used in BayesA. In Figure 3, shrinkage estimation of SNP effects from ridge regression, BayesA, fastBayesA and VanRaden non-linear prediction A (VanRaden A) [7] are plotted against least squares estimates. Comparing with ridge regression, BayesA, fastBayesA and VanRaden A shrink small effects towards zero more than large effects. The estimates from fastBayesA are indistinguishable to that from BayesA for those effects larger than a certain value around 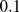 standard deviation and they are close to least squares estimates, but smaller effects are shrunk more heavily toward zero by fastBayesA than BayesA. The reason may be that the local modes of small effects that fastBayesA finds tend to be closer to zero than the mean. This suggests that calculating the mean like VanRaden A instead of mode can be an advantage in some cases since the maximization is over all possible effect values without getting stuck at local modes. Figure 4 shows that in scenarios with 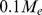 QTL, most of the large effects from fastBayesA tend to be bigger than those from BayesA but similar to those from BayesB, which indicates that with few QTL, the joint mode that fastBayesA finds tend to be larger than BayesA posterior means but close to BayesB posterior means, and that the shrinkage of large effects with fastBayesA is less than with BayesA but similar to BayesB. Furthermore, in scenarios with 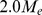 QTL, most of the large effects from fastBayesA are bigger than those from either BayesA or BayesB, indicating that with a large number of QTL, the posterior mode that fastBayesA finds are even larger than posterior means of BayesB. However, Figure 4 also shows that in all four scenarios of genetic architectures, there are subsets of estimated SNP effects that are almost zero with fastBayesA but are large with BayesA and BayesB. The reason might be that for these subsets of SNP effects, fastBayesA chose a mode that is close to zero and is far from the posterior means. This explains the lower accuracy of fastBayesA than BayesB, since some moderately large effects in BayesB are over-shrunk to zero by fastBayesA due to the convergence to a local mode. The above observations suggest that the shrinkage behavior of fastBayesA and the shape of the posterior distribution of SNP effects under the BayesA model require further study.

The regression coefficient of TBV on GEBV was smaller than 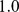 in most scenarios of Dataset A for both fastBayesA and BayesA, which means the variance of GEBV was inflated and GEBV should be shrunk more to make prediction of TBV unbiased [1]. Biases were greater for fastBayesA than BayesA, likely because of insufficient shrinkage of large effects, as shown in Figure 3. Another reason might be that for BayesA residual variance was sampled by MCMC iteration while the simulated real residual variance was used for fastBayesA. The bias for fastBayesA is expected to become smaller than observed here when the residual variance is also updated as mean square error in each step of EM iteration (Appendix S2). This modified algorithm was applied to the 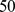 replicates of scenario A1. The average regression coefficient became 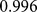 with no change in prediction accuracy.

Each single step of fastBayesA can be regarded as BLUP of breeding values based on a weighted marker-derived relationship matrix. The realized relationship between each pair of individuals not only incorporates information of genome fragments that are IBS or IBD given high density SNP genotypes but also incorporates information about genetic architecture by allowing differing sizes of contributions of each SNP to the overall genetic variance. The relationship matrix used here is similar to the trait-specific relationship matrix in the heuristic TA-BLUP of Zhang et al. [34] but differs in that TA-BLUP used genetic variance as weights for different SNPs. Method fastBayesA and TA-BLUP share the idea that SNPs that are in LD with QTL contribute more to the genetic covariance between individuals for a specific trait than SNPs that are in linkage equilibrium with QTL, but the maximizing behavior of TA-BLUP is not clear. Approximately, TA-BLUP could be regarded as one step of fastBayesA with an improper prior for effect variance, with degrees of freedom and scale parameter close to zero. Yi and Banerjee [23] used degrees of freedom equal to 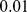 and scale parameter equal to 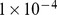 for the prior of effect variance, which resulted in strong shrinkage of small effects. With this choice of hyperparameters, the effect variance of each SNP is dominated by the squared estimated effect and hence for small effects, the effect variance diminishes with EM iteration and the estimated effect is shrunk to zero. Method fastBayesA with such an improper prior was tested on datasets with 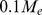 QTL with heterogeneous variance and heritability 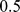, and resulted in much lower prediction accuracy at convergence than in the first several iterations for several replicates (result not shown). This, however, suggests that improper priors, as in Yi and Banerjee [23], can be used to identify the largest effects in genome wide QTL mapping studies but at the risk of decreased predictability for breeding values due to ignoring many small effects.

Method fastBayesA inherits the main advantages that GBLUP possesses and which MCMC-based methods lack. First, animals that have not been genotyped can be included in the model through pedigree relationship using single-step approach by Legarra et al. [35] and Misztal et al. [36], in which phenotypes from ungenotyped animals contribute to the estimates of breeding values and hence marker effects. For MCMC-based methods, genotypes of ungenotyped animals must be imputed in order to include them into the analysis since genotype is indispensible. Second, prediction error variance and hence reliability or accuracy of the GEBV of each animal (especially validation animals) could be obtained using methods by Strandén and Garrick [12]. For MCMC methods, the reliability of GEBV is available only when the posterior distribution of GEBV is known. This requires interim validation during Markov Chain using the sampled SNP effects to calculate the prediction error variance of GEBV.

In conclusion, a fast EM algorithm fastBayesA is shown to approach BayesA estimates of marker effects without requiring MCMC. Simulation studies showed that fastBayesA has similar accuracy to BayesA under a range of genetic architectures. Method fastBayesA can be an appropriate substitute for BayesA for datasets with large numbers of markers or for pedigreed population with ungenotyped animals.

## Supporting Information

Appendix S1 **Expectation of the reciprocal of a scaled inverse Chi-square random variable.**
(PDF)

Appendix S2 **Estimation equations for parameters from fastBayesA.**
(PDF)
