# Supplementary material for: A Fast EM Algorithm for BayesA-Like Prediction of Genomic Breeding Values
Source: PLoS One. 2012 Nov 9;7(11):e49157. doi: 10.1371/journal.pone.0049157 (PMC3494698; doi:10.1371/journal.pone.0049157)
Supplement: Appendix S1 — Expectation of the reciprocal of a scaled inverse Chi-square random variable. (PDF) [file pone.0049157.s001.pdf]

# Appendix S1 Expectation of the reciprocal of a scaled inverse Chi-square random variable

Given that

$$\gamma_j|\sigma_j^2 \sim N(0, \sigma_j^2)$$

and

$$\sigma_j^2 \sim \frac{\nu_{\boldsymbol{\gamma}} S_{\boldsymbol{\gamma}}^2}{\chi_{\nu_{\boldsymbol{\gamma}}}^2},$$

The joint distribution of $\gamma_j$ and $\sigma_j^2$ is

$$\begin{aligned} p(\gamma_j, \sigma_j^2) &\propto \exp\left(-\frac{\nu_{\boldsymbol{\gamma}} S_{\boldsymbol{\gamma}}^2}{2\sigma_j^2}\right)\left(\sigma_j^2\right)^{-\left(1+\frac{\nu_{\boldsymbol{\gamma}}}{2}\right)} \cdot \left(\sigma_j^2\right)^{-\frac{1}{2}} \exp\left(-\frac{\gamma_j^2}{2\sigma_j^2}\right) \\ &\propto \exp\left(-\frac{\nu_{\boldsymbol{\gamma}} S_{\boldsymbol{\gamma}}^2 + \gamma_j^2}{2\sigma_j^2}\right)\left(\sigma_j^2\right)^{-\left(1+\frac{\nu_{\boldsymbol{\gamma}}+1}{2}\right)}. \end{aligned}$$

This is the kernel of the conditional distribution of $\sigma_j^2$ given $\gamma_j$, which is a scaled inverse Chi-square distribution with degrees of freedom $\nu_{\boldsymbol{\gamma}} + 1$ and scale parameter $\frac{\gamma_j^2 + \nu_{\boldsymbol{\gamma}} S_{\boldsymbol{\gamma}}^2}{\nu_{\boldsymbol{\gamma}}+1}$.

To show that

$$\mathbb{E}_{\boldsymbol{\sigma}^2|\boldsymbol{y}, \boldsymbol{\gamma}=\widehat{\boldsymbol{\gamma}}^{(k)}}\left(\frac{1}{\sigma_j^2}\right) = \left(\frac{\{\widehat{\gamma}_j^{(k)}\}^2 + \nu_{\boldsymbol{\gamma}} S_{\boldsymbol{\gamma}}^2}{\nu_{\boldsymbol{\gamma}} + 1}\right)^{-1},$$

it suffices to show that the expectation of the reciprocal of a scaled inverse Chi-square variable is the reciprocal of its scale parameter. Suppose $X$ is a scaled inverse Chi-square random variable with degrees of freedom $\nu$ and scale parameter $S^2$, the probability density function for $X$ is given by

$$p(x|\nu, S^2) = \frac{\left(\frac{\nu S^2}{2}\right)^{\frac{\nu}{2}}}{\Gamma\left(\frac{\nu}{2}\right)} \cdot \frac{\exp\left(-\frac{\nu S^2}{2x}\right)}{x^{1+\frac{\nu}{2}}}.$$

It follows that the probability density function for $Y = \frac{1}{X}$ is

$$\begin{aligned} p(y|\nu, S^2) &= \frac{\left(\frac{\nu S^2}{2}\right)^{\frac{\nu}{2}}}{\Gamma\left(\frac{\nu}{2}\right)} \cdot \frac{\exp\left(-\frac{\nu S^2}{2} y\right)}{\left(\frac{1}{y}\right)^{1+\frac{\nu}{2}}} \cdot \left|-\frac{1}{y^2}\right| \\ &= \frac{\left(\frac{\nu S^2}{2}\right)^{\frac{\nu}{2}}}{\Gamma\left(\frac{\nu}{2}\right)} \cdot \exp\left(-\frac{\nu S^2}{2} y\right) \cdot y^{\frac{\nu}{2}-1}. \end{aligned}$$

This is the probability density function of Gamma distribution with shape parameter $\frac{\nu}{2}$ and rate parameter $\frac{\nu S^2}{2}$. The expectation of Gamma distribution is the shape over rate and therefore the expectation of $\frac{1}{X}$ is $\frac{1}{S^2}$.
