# Supplementary material for: A Fast EM Algorithm for BayesA-Like Prediction of Genomic Breeding Values
Source: PLoS One. 2012 Nov 9;7(11):e49157. doi: 10.1371/journal.pone.0049157 (PMC3494698; doi:10.1371/journal.pone.0049157)
Supplement: Appendix S2 — Estimation equations for parameters from fastBayesA. (PDF) [file pone.0049157.s002.pdf]

Appendix S2 Estimation equations for parameters from fastBayesA

At convergence of the EM algorithm when $\widehat{\boldsymbol{\gamma}}^{(k-1)} \approx \widehat{\boldsymbol{\gamma}}^{(k)}$, the fastBayesA estimates of SNP effects ($\widehat{\boldsymbol{\gamma}}$) and fixed effects ($\widehat{\boldsymbol{\beta}}$) satisfy

$$\widehat{\boldsymbol{\gamma}} = \left[\mathbf{Z}'\mathbf{Z} + \widehat{\mathbf{D}}^{-1}\sigma_e^2\right]^{-1}\mathbf{Z}'\left(\boldsymbol{y} - \mathbf{X}\widehat{\boldsymbol{\beta}}\right),$$

and

$$\widehat{\boldsymbol{\beta}} = \left(\mathbf{X}'\widehat{\mathbf{V}}^{-1}\mathbf{X}\right)^{-1}\mathbf{X}'\widehat{\mathbf{V}}^{-1}\boldsymbol{y},$$

in which

$$\widehat{\mathbf{D}} = \mathrm{diag}\left\{\frac{\widehat{\gamma}_j^2 + \nu_\gamma S_\gamma^2}{\nu_\gamma + 1}\right\}_{j=1}^{m},$$

and

$$\widehat{\mathbf{V}} = \mathbf{Z}\widehat{\mathbf{D}}\mathbf{Z}' + \mathbf{I}\sigma_e^2.$$

In this study the residual variance is assumed known from simulation. In most cases where the residual variance is unknown, the estimate in the kth step of EM iteration is calculated as

$$\{\widehat{\sigma}_e^2\}^{(k)} = \frac{\left[\boldsymbol{y} - \mathbf{X}\widehat{\boldsymbol{\beta}}^{(k)} - \mathbf{Z}\widehat{\boldsymbol{\gamma}}^{(k)}\right]'\left[\boldsymbol{y} - \mathbf{X}\widehat{\boldsymbol{\beta}}^{(k)} - \mathbf{Z}\widehat{\boldsymbol{\gamma}}^{(k)}\right]}{n - \mathrm{rank}(\mathbf{X})}.$$
